# Venetoclax plus dose-adjusted R-EPOCH (VR-DA-EPOCH) or G-EPOCH bridging to subsequent cellular therapy for the patients with transformed lymphoma a single center clinical experience

**DOI:** 10.1007/s00277-024-05618-x

**Published:** 2024-01-22

**Authors:** Shuchao Qin, Rui Jiang, Luomengjia Dai, Yi Miao, Yeqin Sha, Tonglu Qiu, Chongyang Ding, Zhen Wang, Chuanbing Shi, Yi Xia, Lei Fan, Wei Xu, Jianyong Li, Huayuan Zhu

**Affiliations:** 1 Department of Hematology, The First Affiliated Hospital of Nanjing Medical University, Jiangsu Province Hospital, Nanjing, 210029 Jiangsu China; 2 Department of Nuclear Medicine, The First Affiliated Hospital of Nanjing Medical University, Jiangsu Province Hospital, Nanjing, 210029 Jiangsu China; 3 Department of Pathology, The First Affiliated Hospital of Nanjing Medical University, Jiangsu Province Hospital, Nanjing, 210029 Jiangsu China; 4 Department of Pathology, Pukou People’s Hospital, Nanjing, 211800 Jiangsu China

**Keywords:** Indolent lymphoma, Histology transformation, R-EPOCH, Venetoclax

## Abstract

Indolent lymphoma, including chronic lymphocytic leukemia/small lymphocytic lymphoma (CLL/SLL) and follicular lymphoma (FL), can undergo histological transformation into an aggressive subtype, typically diffuse large B-cell lymphoma (DLBCL). The prognosis of transformed lymphoma is poor. In this study, we reported the efficacy and toxicity of a combination of venetoclax, dose-adjusted rituximab or obinutuzumab, etoposide, prednisone, vincristine, doxorubicin, and cyclophosphamide (VR-DA-EPOCH or VG-DA-EPOCH) in 11 patients with biopsy-proven histology transformation into DLBCL, including 8 patients with RT and 3 with transformed FL (tFL). The study was conducted between October 2019 and March 2023 at our single center. The median age of participants at enrolment was 53 years. Six patients (85.7%, 6/7) achieved complete remission (CR) at the end of treatment. The best overall response rate (ORR) and CR rate were both 72.7%, respectively. Two patients received autologous hemopoietic stem cell transplant (ASCT) while two patients received ASCT concurrently with CAR-T therapy for consolidation. With a median follow-up of 13.5 (range, 2.4–29.8) months after enrollment, the median event-free survival, progression-free survival, and overall survival were 9.4, 11.5, and 17.5 months, respectively. Hematologic toxicities of grade ≥3 consisted of neutropenia (90.9%, 10/11), thrombocytopenia (63.6%, 7/11), and febrile neutropenia (54.5%, 6/11). In conclusion, VR-DA-EPOCH or VG-DA-EPOCH was a promising strategy to achieve an early remission, bridging to cellular therapy within this population.

**Supplementary Information:**

The online version contains supplementary material available at 10.1007/s00277-024-05618-x.

## Introduction

Histological transformation to diffuse large B-cell lymphoma (DLBCL) occurs in approximately 2–10% of chronic lymphocytic leukemia/small lymphocytic lymphoma (CLL/SLL) [1] and 15% of follicular lymphoma (FL) [2]. This transformation is always associated with a poor clinical outcome. Patients with clonally related DLBCL-variant Richter transformation (RT) have an unfavorable overall survival (OS), with a median OS of 9–12 months [3–5] while those with transformed FL (tFL) have a median OS of 1–2 years [6]. Due to the unsatisfactory clinical efficacy of standard chemoimmunotherapy (CIT) like R-CHOP (rituximab, cyclophosphamide, doxorubicin, vincristine, and prednisone) or R-CHOP-like regimen in those patients with transformed indolent lymphoma to DLBCL, there is an unmet need for a new strategy [7–10].

Venetoclax, an oral inhibitor of B cell lymphoma-2 (BCL-2), has been authorized for treating relapsed or refractory and frontline CLL/SLL. However, venetoclax as monotherapy in transformed indolent lymphoma showed limited efficacy [11], it has been extensively investigated in combination with standard CIT for this population. The recent addiction of venetoclax to dose-adjusted R-EPOCH (rituximab, etoposide, prednisone, vincristine, doxorubicin, and cyclophosphamide) showed impressive improvement in clinical outcomes for patients with RT and tFL [12, 13]. In this report, we present the efficacy and toxicities of venetoclax when combined with dose-adjusted R-EPOCH (VR-DA-EPOCH) or dose-adjusted G-EPOCH (obinutuzumab, etoposide, prednisone, vincristine, doxorubicin, and cyclophosphamide) in 11 patients with indolent lymphoma transformation.

## Materials and methods

### Patients and data collection

We retrospectively collected the data of 11 patients with VR-DA-EPOCH or venetoclax combined with dose-adjusted G-EPOCH in this study between October 2019 and March 2023 in our center. All patients had a prior history of indolent lymphoma and were confirmed to have transformed into DLBCL histologically, including 8 patients with RT and 3 patients with tFL. Informed consent for publication was obtained from all patients involved in this research.

### Treatment procedures

VR-DA-EPOCH or venetoclax plus dose-adjusted G-EPOCH (VG-DA-EPOCH) was given every 3 weeks as following strategy: venetoclax was administered orally with accelerated ramp-up from 20 mg d1, 50 mg d2, 100 mg d3, 200 mg d4 to 400 mg d5–10 during cycle 1, 400 mg daily at days 1–7 of cycles 2–6, the initial R-EPOCH or G-EPOCH were given as standard rituximab 375 mg/m^2^ on day 0 of cycle 1 and 500 mg/m^2^ on day 0 of cycles 2–6, intravenous obinutuzumab 1000 mg on days 0, 7, and 13 of cycle 1 and day 0 of cycles 2–6, intravenous etoposide 50 mg/m^2^ on days 1–4, vincristine 0.4 mg/m^2^ on days 1–4, doxorubicin 10 mg/m^2^ on days 1–4, cyclophosphamide 750 mg/m^2^ on day 5, and oral prednisone 60 mg/m^2^ daily on days 1–5. Dose adjustments of etoposide, doxorubicin, and cyclophosphamide were based on complete blood count checked twice weekly as reported by Wilson et al. [14]. Prophylaxis was mandatory with granulocyte colony-stimulating factor, acyclovir for herpes viruses, and co-trimoxazole for pneumocystis carinii pneumonia in all patients, and entecavir treatment was prescribed for patients with seropositive occult hepatitis B virus infection (HBsAg negative but HBcAb positive). Cellular therapy was administered in intended patients with complete remission (CR) at end of treatment (EOT).

### Efficacy evaluation and survival analysis

Interim and EOT response assessment was conducted after 2 or 3 cycles and at completion of 6 cycles by contrast-enhanced CT or PET/CT according to 2014 Lugano criteria [15]. Adverse events (AEs) were evaluated according to CTCAE 5.0. Next-generation sequencing (NGS) was conducted in DLBCL samples using our 72-genes B-cell chronic lymphoproliferative disorder panel (Supplementary Table 1). Event-free survival (EFS) was calculated from the date diagnosed as transformation to the date of disease progression and relapse and death, and progression-free survival (PFS) was defined as the time from the date of transformation to progressive disease (PD). OS was calculated from the date of diagnosis to the date of death. Survival analysis was performed by Kaplan-Meier methods.

## Results

### Patient characteristics

The median age of 11 patients at diagnosis of histological transformation was 53 years (35–67), including 8 patients with RT and 3 patients with tFL. Eight of 11 patients (72.7%) received at least one (range, 1–4) prior line treatment for CLL/SLL or FL. Three RT patients received a single-agent ibrutinib treatment (P1, P3, and P6). One RT patient (P8) and two tFL patients (P9 and P10) received CHOP-like treatment in the prior line as indolent lymphoma. Three treatment-naïve patients (P4, P5, and P11) experienced histology transformation during watch and wait. The median SUVmax was 18.5 (range, 4–32.7), and all patients underwent lymph node (*n*=10) or bone (*n*=1) biopsy at the site of SUVmax or secondary SUV uptake (inaccessible for the highest SUVmax site). 11 patients were diagnosed as DLBCL immunohistochemically, including 4 patients confirmed as double expression (BCL-2 and MYC) DLBCL and one patient (P10) confirmed as triple hit lymphoma (THL) by further analysis of fluorescence in situ hybridization (FISH). MYC amplification was further detected in circulation tumor DNA by next generation sequence in P2 and P8, without MYC or BCL-2 translocation. Among 8 patients with RT, 7 available patients were further confirmed as clonally related RT to prior CLL, including one patient presented two pathogenetic clones and two patients (P3, P5) with stereotype subset 8. The clinical characteristics of 11 patients were summarized in Table 1 and the detailed clinical characteristics were listed in Supplementary Table 2–4.

**Table 1:** Baseline characteristics at enrollment of the regimen

All patients (*n*=11)	
Sex	
Male	5/11 (45.5%)
Female	6/11 (54.5%)
Median age (years)	53 (range, 35–67)
ECOG performance status	
0-1	6/11 (54.5%)
2-3	5/11 (45.5%)
Histology	
GCB	3/11 (27.3%)
Non-GCB	8/11 (72.7%)
Bulky lymphadenopathy > 5 cm	5/11 (45.5%)
Extranodal involvement	7/11 (63.6%)
Bone marrow involvement	3/11 (27.3%)
Median number of prior therapies	1 (range, 0–4)
Baseline blood count at enrollment	
Median white blood cell count (×10^9^/L)	6.52 (range, 1.73–88.4)
Median absolute neutrophil count (×10^9^/L)	3.2 (range, 1.3–6.4)
Median hemoglobin (g/L)	114 (range, 74–163)
Median platelets (×10^9^/L)	192 (range, 51–403)
Median LDH (U/L)	346 (range, 159–1186)

### Treatment disposition and response

**Fig. 1:**
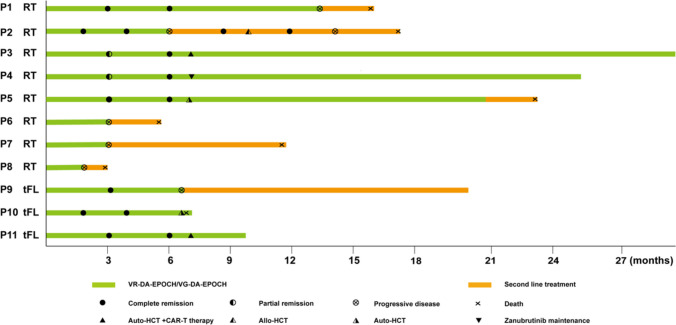
Treatment scheme of the 11 patients

**Fig. 2:**
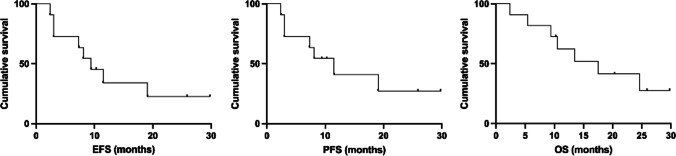
Event-free survival, progression-free survival (PFS), and overall survival (OS) for 12 patients.

All patients received at least 2 cycles of VR-DA-EPOCH/VG-DA-EPOCH and were available for response assessment. The interim overall response rate (ORR) and CR rate was 72.7% (8/11) and 54.5% (6/11), respectively. Four patients (P6, P7, P8, and P9) progressed early, failed to accomplish the planned six cycles of the regimen and transmitted to second line therapy. At EOT, in six of seven available patients (85.7%, 6/7) achieving CR, one patient (P2) progressed at EOT assessment, attained second CR with two cycles of chidamide and sintilimab combined with polatuzumab vedotin, underwent allogeneic hematopoietic stem cell transplantation (allo-HCT) from matched unrelated donor, experienced grade 1 of dermatological graft rejection, progressed post 168 days, and ceased four months later. The best ORR and CRR were both 72.7%. Four of seven were candidates for cellular therapy as consolidation. P3 and P11 were consolidated by autologous hematopoietic stem cell transplantation (ASCT) concurrently with CD19-CAR-T therapy and remained in CR with a follow-up of 29.8 and 10.3 months. Two patients underwent ASCT: P5 progressed post 259 days and transmitted to zanubrutinib but ceased 5.5 months later while P10 ceased due to complications after stem cell transplant. Two patients did not receive cellular therapy as consolidation: one (P4) received zanubrutinib as maintenance and remained CR with a follow-up of 25.9 months while the other patient (P1) progressed 6 months after EOT and received rituximab, gemcitabine and oxaliplatin (R-GemOx) as bridge therapy but ceased with a follow-up of 13.5 months (Fig. 1). With a median follow-up of 13.5 (range, 2.4–29.8) months after enrollment, the median EFS, PFS, and OS were 9.4, 11.5, and 17.5 months, respectively (Fig. 2).

### Adverse events

The median number of cycles was 6 (range, 2–6), and the median dose intensity was 60% (50–90%) of standard EPOCH and 400 mg daily with 5 consecutive days for venetoclax. Two patients (P6 and P7) started initially at less than 50% dose dense of EPOCH due to age and poor ECOG performance status (ECOG-PS), failed to escalate within subsequent cycles due to febrile neutropenia. Five patients experienced at least one time of dose de-escalation of EPOCH due to hematologic toxicities. The most frequent grade 3 or 4 hematological AEs were neutropenia (90.9%, 10/11), thrombocytopenia (63.6%, 7/11) and febrile neutropenia (54.5%, 6/11), and non-hematological AEs were sepsis (18.2%, 2/11), diarrhea (9.1%, 1/11), pulmonary infection (9.1%, 1/11), fibrinogen decrease (9.1%, 1/11), diarrhea (9.%, 1/11), nausea (9.1%, 1/11), and vomit (9.1%, 1/11). Grade 1 or 2 hematological AEs were anemia (18.2%, 2/11) and thrombocytopenia (9.1%, 1/11) and non-hematological AEs were fatigue (27.3%, 3/11), paresthesia (18.2%, 2/11), alanine aminotransferase increase (18.2%, 2/11), aspartate aminotransferase (18.2%, 2/11), and hypokalemia (18.2%, 2/11). The efficacy, response, and grad 3–4 AEs of each patient are listed in Table 2.

**Table 2:** The response and tolerance of VR-EPOCH

	Cycles completed	Best response	PFS (months)	OS (months)	AEs	
					Hematological AEs of grades 3–4 (No. of events/cycles)	Non-hematological AEs of grades 3–4 (No. of events/cycles)
P1	6	CR	11.5	13.5	Neutropenia (5/6) Febrile neutropenia (3/6) Thrombocytopenia (1/6)	Sepsis(2/6)
P2	6	CR	7.3	17.5	Neutropenia (6/6) Febrile neutropenia (2/6)	Sepsis (1/6) Pulmonary infection (1/6)
P3	6	CR	NR	NR	Neutropenia (3/6)	
P4	6	CR	NR	NR	Neutropenia (6/6) Anemia (4/6) Thrombocytopenia (1/6)	Diarrhea (1/6)
P5	6	CR	19.1	24.6	Neutropenia (6/6) Anemia (3/6) Thrombocytopenia (1/6) Febrile neutropenia (1/6)	
P6	3	PD	3	5.4	Neutropenia (3/3) Anemia (2/3) Thrombocytopenia (3/3)	
P7	3	PD	3	7.5	Neutropenia (3/3) Thrombocytopenia (3/3) Febrile neutropenia (2/3)	Nausea (1/3) Vomit (1/3)
P8	2	SD		2.4	Neutropenia (2/2) Anemia (1/2) Thrombocytopenia (1/2) Febrile neutropenia (1/2)	
P9	4	CR	8.1	NR	Neutropenia (4/4) Febrile neutropenia (4/4)	Fibrinogen decreased (1/3)
P10	4	CR	NR	9.4	Neutropenia (4/4) Thrombocytopenia (2/4)	
P11	4	CR	NR	NR		

*AEs* adverse events, *CR* complete remission, *NR* not reached, *OS* overall survival, *PD* progressive disease, *PFS* progression-free survival, *SD* stable disease

## Discussion

Histological transformation occurs in approximately 2–15% of patients with CLL/SLL [16] and 15% of patients with FL [17–19]. Numerous factors were reported to be associated with an increased risk of transformation in indolent lymphomas. In CLL/SLL, TP53 aberrations, loss of CDKN2A gene, NOTCH1 mutations, unmutated immunoglobulin heavy chain variable region (IGHV) status, and stereotyped BCR subset 8 at diagnosis were reported to be associated with RT development [20–23]. Meanwhile, in FL, advanced stage and high Follicular Lymphoma International Prognostic Index (FLIPI) scores at diagnosis have been reported to increase the risk of tFL [17, 18]. Furthermore, several genomic and biological features were relevant to the heightened susceptibility of developing tFL, including mutations in TP53, CDKN2A/B, MYC, and B2M, as well as chromosome abnormalities such as del(11q), del(6q), +2, +3q and +5 [24]. The clinical and genetic characteristics of the patients in our small cohort were concordant with previous studies. Among 8 patients with a history of CLL/SLL, 7 patients who were available for IGHV analysis had unmutated IGHV at CLL/SLL stage and were confirmed as clonal-related RT, 2 of them with stereotyped BCR subset 8. Previous research suggests that gene alterations involving DNA damage response and cell cycle, such as TP53 and CDKN2A/B may lead to genetic instability and dysregulation of cell proliferation, thus potentially contributing to the development of histological transformation [24–26]. TP53 abnormalities were identified in five patients after histological transformation, two of whom (P1 and P9) had TP53 alterations during the preceding indolent lymphoma phase. Furthermore, we detected a comparable proportion of complex karyotype (CK) at diagnosis of transformation with a rate of 50% as reported before that CK was associated with the increased risk of histological transformation [27].

The optimal treatment for histological transformation of indolent lymphoma remains unclear. Prior to the era of Bruton tyrosine kinase inhibitors (BTKi), traditional chemotherapy regimens such as R-CHOP and R-EPOCH yielded unsatisfactory outcomes for both relapsed/refractory and transformed follicular lymphoma [9, 10, 28–34].The emergence of novel targeted agents including BTKi and Bcl-2 inhibitor venetoclax brought about novel strategies has introduced new treatment strategies for this patient population; however, ORR and CR rates achieved with single-agent therapy were only approximately 40–75% and 10–25%, respectively [11, 35–39]. Consequently, new strategies are eagerly awaited. Multiple studies have shown that a combination of the BCL-2 inhibitor venetoclax and CIT is a promising regimen. In a cohort of patients with RT, the combination of venetoclax and R-EPOCH induced deep remission with an ORR of 62% (16/26) and a CR rate of 50% (13/26). 11 patients (84.6%) achieved undetectable minimal residual disease (UMRD) in bone marrow (BM), and 9 patients were able to undergo cell therapy or allo-HCT. With a median follow-up of 17 months, the median PFS and OS was 10.6 and 19.6 months, respectively [12]. The CAVALLI study investigated the addition of venetoclax to R-CHOP in non-Hodgkin lymphoma (NHL) including patients with transformed lymphoma. In the phase 2 proportion of the CAVALLI study, the addition of venetoclax to R-CHOP showed superiority of survival and response in comparison with R-CHOP in patients with BCL-2^+^ disease, with a CR rate of 64% and a 2-year PFS of 78% [40]. However, the optimal choice of subsequent cellular therapy, including ASCT, allo-HCT and CAR-T-cell therapy remains uncertain despite the promising result from several studies. In a registry study of patients with DLBCL-RT, comparable survival benefit was observed between ASCT and allo-HCT recipients, with a 3-year PFS of 48% and 43%, respectively [41]. However, the risk of allo-HCT such as graft-versus-host disease (GVHD) and non-relapse mortality (NRM) may restrict its widespread use. Furthermore, CD19-CAR-T cell therapy exhibits remarkable efficacy, achieving a CR rate of 67.5% in RT and 62% in tFL [42, 43]. Despite superior clinical activity, further investigation is necessary to determine the sustained remission of CAR-T cell therapy. Recently, CD19/CD22 CAR-T cell therapy following ASCT showed a striking efficacy and manageable toxicity in a prospective study of aggressive B-cell lymphoma, including seven patients with tFL. 85.7% patients achieved CR at 6 months post-treatment, and the 2-year PFS rate was 83.3% [44].

Here, response rate of VR-DA-EPOCH or VG-DA-EPOCH was also highly encouraging, with the best CR rate of 72.7%. Eight patients achieved an early response, including three with TP53 mutation. Of note, among seven patients with BCL2 overexpression assessed by immunohistochemistry (IHC) of biopsy tissue, five (71.4%) patients achieved CR as best response, including one patient with triple-hit lymphoma. This result is consistent with that found in the CAVALLI study. Although the cohort sample size was small, CK remained an unfavorable factor for the regimen as all five patients showed progression, as similarly reported [12, 45, 46]. Furthermore, patients with TP53 abruptions had comparable outcomes to those without, consistent with previous reported [12]. The toxicity of myelosuppression was significant, with 10 patients experiencing neutropenia of grades 3–4 at least once. However, it was controllable with the use of G-CSF support and dose adjustment. Further investigation is required to optimize venetoclax and R-EPOCH or G-EPOCH doses and durations to enhance the response while managing toxicity. Cellular therapy should be considered for consolidation in patients with histological transformation, particularly if they have TP53 aberrations. The presence of del(17p) and previous receipt of novel agents did not affect the prognosis for allo-HCT [41]. However, the incidence of GVHD and NRM limited the widespread use of allo-HCT. This highlights the need for a less intensive and more potentially curative regimen. In a study prospectively examining the application of ASCT in conjunction with CD19/CD22 CAR T therapy for aggressive lymphoma, patients with TP53 aberrations exhibited a 2-year PFS rate of 84.6%. The treatment demonstrated impressive efficacy and a survival benefit across different genetic subtypes, including TP53 aberrations and MYC rearrangement [44]. In our cohort, patients with ongoing remission experienced less prior treatment while those with progression presented unfavorable biological features. However, follow-up needs to be extended for these patients to better observe PFS. The optimal choice of subsequent cellular therapy as consolidation to improve antitumor activity and reduce relapse incidence was still an unmet need based on our study. Achieving long-term remission may require an early deep remission followed by ASCT in combination with CAR-T cell therapy as consolidative treatment. The optimal management of patients with histological transformation of indolent lymphoma remains an unanswered question, and individually comprehensive management is required due to the disease’s heterogeneity concerning age, comorbidities, performance status and genetic features.

In conclusion, our cohort demonstrated that combining venetoclax with R-DA-EPOCH or G-EPOCH was a promising strategy to achieve early remission and bridge to cellular therapy. In managing transformed indolent lymphoma, therapies that achieve deep response, such as VR-DA-EPOCH or VG-DA-EPOCH as a bridge, should be considered comprehensive strategies leading to subsequent cellular treatment towards curative survival. Further research is required to develop prognostic factors and models for disease recurrence.

### Supplementary information

ESM 1 (DOCX 37 kb)

## Data Availability

The datasets generated during and/or analyzed during the current study are available from the corresponding author on reasonable request.
