# Supplementary material for: Venetoclax plus dose-adjusted R-EPOCH (VR-DA-EPOCH) or G-EPOCH bridging to subsequent cellular therapy for the patients with transformed lymphoma a single center clinical experience
Source: Ann Hematol. 2024 Jan 22;103(5):1635–42. doi: 10.1007/s00277-024-05618-x (PMC11009738; doi:10.1007/s00277-024-05618-x)
Supplement: Supplementary file 1 — (DOCX 37 kb) [file 277_2024_5618_MOESM1_ESM.docx]

**Supplementary material**

**Supplementary Table 1** The 72-genes chronic B-lymphoproliferative disorder panel

| ARID1A | ARID2 | ATM | ATP6AP1 | ATP6V1B2 |
| --- | --- | --- | --- | --- |
| B2M | BCL11A | BCL2 | BCOR | BIRC3 |
| BRAF | BTK | CARD11 | CCND1 | CD58 |
| CDKN1B | CDKN2A | CDKN2B | CHD2 | CREBBP |
| CXCR4 | DDX3X | EGR2 | EP300 | EZH2 |
| FAS | FAT1 | FBXW7 | FOXO1 | FUBP1 |
| IGLL5 | IKBKB | KLF2 | KLHL6 | KMT2C |
| KMT2D | KRAS | MAP2K1 | MAP3K14 | MED12 |
| MGA | MYC | MYCN | MYD88 | NFKBIA |
| NFKBIE | NOTCH1 | NOTCH2 | NRAS | PLCG2 |
| POT1 | PTPN11 | PTPRD | RB1 | REL |
| RPS15 | RRAGC | SAMHD1 | SETD2 | SF3B1 |
| SMARCA4 | SPEN | TET2 | TNFAIP3 | TNFRSF10A |
| TNFRSF10B | TNFRSF14 | TP53 | TRAF2 | TRAF3 |
| UBR5 | XPO1 |  |  |  |

**Supplementary Table 2** Clinical characteristics of eight patients at diagnosis of chronic lymphocytic leukemia/small lymphocytic lymphoma (CLL/SLL)

|  | Gender | Age at CLL/SLL diagnosis | Lugano  stage | Rai/Binet  stage | IGHV | TP53 mutation | Cytogenetic abnormalities | Complex karyotype | NOTCH1 mutation | Prior therapy for CLL/SLL | Time from CLL/SLL diagnosis to progression (months) | |
| --- | --- | --- | --- | --- | --- | --- | --- | --- | --- | --- | --- | --- |
| P1 | M | 43 | IV | / | UM,  4-39*01 | Yes | del(13q14) | NA | No | I | 12.2 |  |
| P2 | M | 50 | / | III/C | UM  4-39*01 | No | del(11q) | 45, XY, del(2) (q33), der(5), del(9) (p13), del(11) (q23), add(14)(q23), -21[1]/ 46, XY, add(2) (p25), del(11) (q23), add(14) (q32)[1]/ 46,XY[8] | No | iFCR | 11.6 |  |
| P3 | F | 64 | / | II/B | UM  4-39*01 | No | del(13q14)  del(17p) | No | Yes | I | 6.7 |  |
| P4 | F | 56 | IV | / | UM  1-3*01 | No | +12 | No | Yes | / | 30.8 |  |
| P5 | F | 52 | / | II/B | UM  4-39*01 | No | del(6q23)  +12 | 47, XX, +12, t(14;19) (q32;q13), der(19) t(14;19) add(19) (p11)[5] /58, XX, +2, +2, +4,der(4) t(1:4) (q25;q35) *2, +5, +11, +12, +12,+13,+14, t(14;19) (q32;q13)*2, +17, +19, der(19) t(14;19) add(19) (p11)*2, +22[1] /79, XXX, der(4) t(1;4)(q25;q35)*2, +5, +12, +12, t(14;19) (q32;q13)*2，+15,+16,+17,+18, +18, der(19) t(14; 19) add(19) (p11)*2, +20, +22[1] | Yes | / | / |  |
| P6 | F | 66 | / | IV/C | UM  4-39*01 | No | NA | 46, XX, t(2;14) (p13;q12)[2]/ 46, idem, del(9) (p12) [12]/ 46, XX, del(11) (q14)[1] | No | I | 17 |  |
| P7 | M | 54 | IV | / | UM  3-28*01 | NA | del(11q)  del(13q14) | NA | NA | FC, FCR, I, HMPL-523^$^, ChR | 56 |  |
| P8 | F | 38 | IV | / | NA | NA | NA | NA | NA | R^2^-CHOP, BR, I | 20 |  |

^$^: spleen tyrosine kinase (SYK) inhibitor

CHOP: cyclophosphamide, doxorubicin, vincristine, prednisone; Ch:chlorambucil; ChR: chlorambucil, rituximab; F: female; FC: fludarabine, cyclophosphamide; FCR: fludarabine, cyclophosphamide and rituximab; I: ibrutinib; iFCR: ibrutinib, fludarabine, cyclophosphamide and rituximab; IGHV: immunoglobin heavy chain; M: male; F:female; NA: not available; R-CHOP: rituximab, cyclophosphamide, doxorubicin, vincristine, prednisone; R^2^-CHOP: rituximab, lenalidomide, cyclophosphamide, doxorubicin, vincristine, prednisone; RS: Richter syndrome; UM: unmutated.

**Supplementary Table 3** Clinical characteristics of 3 patients at follicular lymphoma (FL) stage

|  | Gender | Age at FL diagnosis | Ann Arbor  stage | B symptoms | FLIPI score | ECOG performance score | Extranodal involvement | SUVmax | FL grade | TP53 mutation | Other gene mutation | Prior therapy for FL | Time from FL diagnosis to progression (months) |
| --- | --- | --- | --- | --- | --- | --- | --- | --- | --- | --- | --- | --- | --- |
| P9 | M | 50 | IV | Yes | 4 | 1 | BM, pleura | 19 | 3B | Yes | MYD88, BCL2, KMT2D, CREEP, TNFAIP3, TRAF2, BCOR, KLHL6 | R2-CHOP | 16.43 |
| P10 | F | 50 | IV | Yes | 2 | 0 | BM | NA | II | NA | NA | R-CHOP | 77.9 |
| P11 | M | 33 | IV | No | 2 | 0 | Bone | 7.57 | II | NA | NA | No | 20.7 |

BM: bone marrow; FL: follicular lymphoma

**Supplementary Table 4** Clinical characteristics of 11 patients at diffuse large B cell lymphoma (DLBCL) stage

|  | Age | ECOG  performance score | Ann Arbor stage | B  symptoms | IPI | Bulky disease  (long axis ≥ 5 cm) | SUVmax | Complex karyotype  (sample) | Serum  LDH (U/L) | DLBCL type  (by IHC) | Bcl-2 expression^✻^ | C-myc  expression^✻^ | Extranodal presentation |
| --- | --- | --- | --- | --- | --- | --- | --- | --- | --- | --- | --- | --- | --- |
| P1^#^ | 44 | 2 | III | Yes | 3 | Yes^#^ | 13.2 | 40,XY,-4,der(5),der(6),-10,-13,-13,-14,-15,-20,+mar[1]/46,XY[5]  (PB) | 315 | Non-GCB | NA | NA | None |
| P2 | 51 | 2 | IV | Yes | 3 | No | 13.2 | 40,XY,-4,der(5),der(6),-10,-13,-13,-14,-15,-20,+mar[1]/46,XY[5]  (PB) | 560 | Non-GCB | ＞75%+ | ＞60%+ | Bone |
| P3 | 64 | 2 | III | Yes | 3 | No | 12.8 | No | 159 | Non-GCB | 70%+ | 60%+ | None |
| P4^#^ | 59 | 1 | IV | No | 2 | Yes^#^ | 31.5 | No | 469 | Non-GCB | ＞90%+ | NA | None |
| P5 | 52 | 0 | IV | Yes | 2 | No | 4 | 47, XX, +12, t(14;19) (q32;q13), der(19) t(14;19) add(19) (p11)[5] /58, XX, +2, +2, +4,der(4) t(1:4) (q25;q35) *2, +5, +11, +12, +12,+13,+14, t(14;19) (q32;q13)*2, +17, +19, der(19) t(14;19) add(19) (p11)*2, +22[1] /79, XXX, der(4) t(1;4)(q25;q35)*2, +5, +12, +12, t(14;19) (q32;q13)*2，+15,+16,+17,+18, +18, der(19) t(14; 19) add(19) (p11)*2, +20, +22[1] (BM) | 984 | Non-GCB | + | 30%+ | BM |
| P6 | 67 | 2 | III | No | 3 | No | 32.7 | NA | 346 | Non-GCB | 20%+ | 50%+ | None |
| P7^#^ | 59 | 2 | IV | Yes | 3 | Yes^#^ | 12.8 | 77-82, XXY, 2p-*2, der(11),-12,-13,-14,-14,-15,-15,-17,-17,-21,22q-,[inc4][cp4] (BM) | 255 | Non-GCB | 90%+ | 10%+ | BM |
| P8^#^ | 39 | 3 | IV | Yes | 4 | Yes^#^ | 28.3 | No | 743 | Non-GCB | 80%+ | 70%+ | Bone, BM, stomach, pleura, pancreas, kidney |
| P9 | 51 | 0 | IV | Yes | 4 | No | 19 | 46,XY,del(13)(q14q22)[1]/46,XY[14] (BM) | 173 | GCB | + | 50%+ | Urinary system, pleura, lung |
| P10 | 53 | 0 | IV | No | 3 | No | 18.5 | 46, XX[15] (BM) | 166 | GCB | 80%+ | 40%+ | Stomach |
| P11^#^ | 35 | 0 | IV | No | 4 | Yes | 21.12 | 46, XY[20] (BM) | 1186 | GCB | 80%+ | 60%+ | Pleura |

^#^: 5 patients (P1, P4, P7, P8 and P11) presented bulky lymphadenopathy with the maximum long axis ≥ 10 cm. ^✻^: The Bcl-2 and C-myc expression were detected by immunohistochemistry of biopsy tissue.

DLBCL: diffuse large B cell lymphoma; IHC: immunohistochemistry; IPI: international prognostic index; LDH: lactate dehydrogenase; non-GCB: non germinal center B-cell; PB: peripheral blood; SUV: standard uptake value.
